# Biologics for rheumatoid arthritis: an overview of Cochrane reviews

**DOI:** 10.1590/S1516-31802010000500013

**Published:** 2010-09-02

## Abstract

**BACKGROUND:**

The biologic disease-modifying anti-rheumatic drugs (DMARDs) are very effective in treating rheumatoid arthritis (RA), however there is a lack of head-to-head comparison studies.

**OBJECTIVES:**

To compare the efficacy and safety of abatacept, adalimumab, anakinra, etanercept, infliximab, and rituximab in patients with RA.

**METHODS:**

This ‘Overview of Reviews’ was done by including all Cochrane Reviews on Biologics for RA available in The Cochrane Library. We included only data on standard dosing regimens for these biologic DMARDs from placebo-controlled trials. The primary efficacy and safety outcomes were ACR50 and withdrawals due to adverse events. We calculated Risk Ratios (RR) for efficacy, Odds Ratio (OR) for safety and combined estimates of events across the placebo groups as the expected Control Event Rate (CER). Indirect comparisons of biologics were performed for efficacy and safety using a hierarchical linear mixed model incorporating the most important study level characteristic (i.e. type of biologic) as a fixed factor and study as a random factor; reducing the between study heterogeneity by adjusting for the interaction between the proportion of patients responding on placebo and the duration of the trial.

**MAIN RESULTS:**

From the six available Cochrane reviews, we obtained data from seven studies on abatacept, eight on adalimumab, five on anakinra, four on etanercept, four on infliximab, and three on rituximab. The indirect comparison estimates showed similar efficacy for the primary efficacy outcome for all biologics with three exceptions. Anakinra was less efficacious than etanercept with a ratio of RRs (95% CI; P value) of 0.44 (0.23 to 0.85; P = 0.014); anakinra was less efficacious than rituximab, 0.45 (0.22 to 0.90; P = 0.023); and likewise adalimumab was more efficacious than anakinra, 2.34 (1.32 to 4.13; P = 0.003). In terms of safety, adalimumab was more likely to lead to withdrawals compared to etanercept, with a ratio of ORs of 1.89 (1.18 to 3.04; P = 0.009); anakinra more likely than etanercept, 2.05 (1.27 to 3.29; P = 0.003); and likewise etanercept less likely than infliximab, 0.37 (0.19 to 0.70; P = 0.002).

**AUTHORS’ CONCLUSIONS:**

Based upon indirect comparisons, anakinra seemed less efficacious than etanercept, adalimumab and rituximab and etanercept seemed to cause fewer withdrawals due to adverse events than adalimumab, anakinra and infliximab. Significant heterogeneity in characteristics of trial populations imply that these finding must be interpreted.

**This review should be cited as:**

Singh JA, Christensen R, Wells GA, Suarez-Almazor ME, Buchbinder R, Lopez-Olivo MA, Tanjong Ghogomu E, Tugwell P. Biologics for rheumatoid arthritis: an overview of Cochrane reviews. Cochrane Database of Systematic Reviews 2009, Issue 4. Art. No.: CD007848. DOI: 10.1002/14651858.CD007848.pub2.

**FURTHER INFORMATION:** Centro Cochrane do Brasil Rua Pedro de Toledo, 598 Vila Clementino — São Paulo (SP) — Brasil CEP 04039-001 Tel. (+55 11) 5579-0469/5575-2970 http://ww.centrocohranedobrasil.org.br/

**This section was edited under the responsibility of the Brazilian Cochrane Center**

Full review is available (free access) from: http://www.cochranejournalclub.com/biologics-for-rheumatoid-arthritis-clinical/pdf/CD007848.pdf

## COMMENTS

The biological disease-modifying antirheumatic drugs (DMARDs) cited in this systematic review on treatments for rheumatoid arthritis have been approved by the Food and Drug Administration (FDA) in the United States and by the Brazilian Health Surveillance Agency (ANVISA) whenever at least one non-biological DMARD (methotrexate, hydroxychloroquine, leflunomide, sulfasalazine or minocycline) has failed or been ineffective in attempts to control the inflammatory activity. Biological DMARDs may or may not be used in association with non-biological DMARDs, except for rituximab, which is indicated after previous use of another biological DMARD has failed or been ineffective and therefore is indicated for cases of greater severity. Anakinra is not available in Brazil (used in < 5% of rheumatoid arthritis cases using biological DMARDs in the United States). All are equally effective when compared with placebo. It would be desirable if there were significant studies that made comparisons between the biological agents ("head-to-head"). Among the studies discussed in this review, the lack of uniformity among them with regard to disease severity, duration, prognostic criteria present and type and length of previous use of non-biological DMARDs were noteworthy.

Regarding the safety of these drugs, all of them, without exception, have potential adverse effects (mainly facilitating the emergence of infections). There are still no consistent conclusions regarding the potential for development of malignancy.

Currently, treatment of rheumatoid arthritis with biological agents should be indicated based on the individual characteristics of each rheumatoid arthritis patient (aggressiveness of the disease, prognostic factors, sequelae, comorbidities etc.); on the cost-effectiveness of the treatment; and, especially, on the consensus that has been reached with the patient, after extensive discussion about the possible benefits, side effects and risks from the treatment.^1,2^
